# Pheochromocytoma of the urinary bladder: a systematic review of the contemporary literature

**DOI:** 10.1186/1471-2490-13-22

**Published:** 2013-04-29

**Authors:** Jonathan A Beilan, Adrienne Lawton, Julio Hajdenberg, Charles J Rosser

**Affiliations:** 1 Section of Urologic Oncology, MD Anderson Cancer Center Orlando, 1400 S. Orange Ave, Orlando, FL 32806, USA; 2 College of Medicine, University of Central Florida, Orlando, FL 32827, USA; 3 Department of Pathology, Orlando Health/MD Anderson Cancer Center Orlando, Orlando, FL 32806, USA; 4 Section of Genitourinary Oncology, MD Anderson Cancer Center Orlando, Orlando, FL 32806, USA

**Keywords:** Paraganglioma, Pheochromocytoma, Bladder, Treatment, Diagnosis, Prognosis

## Abstract

**Background:**

Pheochromocytoma (paraganglioma) of the urinary bladder is a rare tumor. Herein we sought to review the contemporary literature on pheochromocytomas of the urinary bladder in order to further illustrate the presentation, treatment options and outcomes of patients diagnosed with these tumors.

**Methods:**

A comprehensive review of the current literature was conducted according to the PRISMA guidelines by accessing the NCBI PubMed database and using the search terms “paraganglioma, pheochromocytoma, bladder.” This search resulted in the identification of 186 articles published between January 1980 and April 2012 of which 80 articles were ultimately included in our analysis.

**Results:**

Pheochromocytomas usually occurred in young adult Caucasians (mean age, 43.3 years; range,11–84 years). According to the literature, the most common symptoms and signs of pheochromocytomas of the urinary bladder were hypertension, headache, and hematuria. Of the 77 cases that commented on catecholamine production, 65 patients had biochemically functional tumors. Approximately 20% of patients were treated by transurethral resection alone, 70% by partial cystectomy and 10% by radical cystectomy. The 75 patients with follow-up information had a mean follow-up of 35 months. At the time of last follow-up, 15 (14.2%) had disease recurrence, 10 (9.4%) had metastasis, and 65 (61.3%) were alive.

**Conclusions:**

Pheochromocytomas of the urinary bladder tend to be functional and occur mostly in young adult Caucasians. Patients with localized tumors have an extremely favorable prognosis and may be managed by less aggressive modalities, whereas patients with metastatic disease have a significant reduction in survival rates despite aggressive treatment.

## Background

Pheochromocytoma of the urinary bladder is a rare tumor that originates from chromaffin tissue of the sympathetic nervous system associated with the urinary bladder wall. Pheochromocytomas are tumors of the sympathetic nervous tissue and may be non-functional or functional, *i.e.,* secrete catecholamine causing paroxysmal hypertension, palpitation, and micturition syncope [1]. Typically these tumors possess the capacity to invade and thus are deemed malignant, yet lack mitoses and cellular dissociation that are usually associated with malignant tumors [2]. Numerous, small series case reports have been published in the English literature since it was first reported in 1953 by Zimmerman *et al.*[3]. Herein we sought to review the contemporary literature on pheochromocytoma of the urinary bladder in hopes of further clarifying presentation, treatment options and outcomes of patients with pheochromocytomas of the urinary bladder.

## Methods

**Figure 1:**
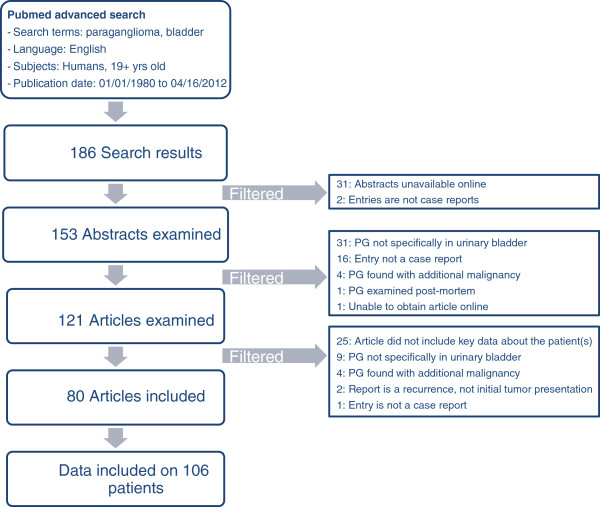
Literature search schema.

A review of the current literate was conducted by accessing the NCBI PubMed database (http://www.ncbi.nlm.nih.gov/pubmed). Filters applied in an advanced search included: the search terms of “paraganglioma, pheochromocytoma, bladder”; English language; human subjects 19 years of age and older; publication dates of January 1980 to April 2012. Figure 1 illustrates the work-flow of the comprehensive literature review, which adhered to the PRISMA systematic review guidelines [4]. This search delivered 186 results from which 153 abstracts were reviewed. A total of 121 articles were ultimately reviewed in full. Patient characteristics were divided into “primary” and “secondary” demographics for ease of data recording. Reasons for exclusion included missing any primary demographic (patient age, sex, presenting symptoms, type of surgery) or more than three secondary demographics (patient race, catecholamine levels, tumor size, tumor grade and stage, last follow up). Due to failure to report primary demographics and/or secondary demographics, an additional 25 articles eventually were excluded from final analysis. Thus the final analysis comprised 80 articles that reported on 106 patients. Relevant clinical, pathologic, laboratory, radiologic and follow-up data from these 80 articles were collected in a database, which allowed the reporting of descriptive statistics.

## Results

Eighty articles on pheochromocytoma of the urinary bladder were identified on Pubmed and were incorporated in our analysis, which included 106 patients [4-82]. The demographics and presenting symptomology are summarized in Table 1. The mean patient age was 43.3 years (range, 11–84 years). The male-to-female ratio was 1.07 to 1. The most common symptoms were hypertension (54.7%), headache (48.1%), hematuria (47.2%) and syncope/palpitations (43.4%). Micturition attacks were reported in 52.8% of patients.

**Table 1:** Demographics and presenting symptoms

**Variable**	**N (%)**
**Total Patients**	106
**Sex**	
Males	55
Female	51
**Age**	
Mean age (years)	43.3
Age range (years)	11-84
**Presenting symptom(s)**	
Micturition attacks	56 (52.8)
Hypertension	58 (54.7)
Headache	51 (48.1)
Hematuria	50 (47.2)
Syncope/palpitations	46 (43.4)
Diaphoresis	21 (19.8)
Micturition disturbances (e.g., urgency, dysuria)	11 (10.4)
Dizziness	10 (9.4)
Abdominal/flank pain	6 (5.7)
Dyspnea/chest pain	5 (4.7)
Malaise	3 (2.8)
Incidental finding	3 (2.8)
Other	11 (10.4)
**Catecholamines**	
↑ VMA, metanephrine, or catecholamines	65 (61.3)
Unknown/not mentioned	29 (27.4)
**Tumor size**	
Mean (cm)	3.9
Median (range) (cm)	3.45 (1.0-9.1)
Unknown/not mentioned	26 (24.5)

Average tumor size was 3.9 cm (median 3.45, range 1–9.1 cm). Few studies (n = 7) reported exact tumor stage by the TNM criteria. Of the 106 patients, 65 (61.3%) were noted to have a functional paraganglioma as evident by elevated VMA, metanephrine and/or catecholamines. Furthermore, limited articles reported on CD56, Chromagranin A, and synaptophysin immunohistochemical staining.

The most commonly reported treatment for patients with paraganglioma of the urinary bladder was partial cystectomy 73 (68.9%). Other treatment options for localized/locally advanced paraganglioma of the urinary bladder include TURBT (21; 19.8%) and radical cystectomy (12; 11.3%). Thirty-one patients included in this study did not have known follow-up. Mean follow of the remaining cohort was 34.6 months (median 18; range 0.75-372 months). Fifteen patients (14.2%) were noted to develop a recurrence of the tumor, while 60 patients (56.6%) had no evidence of disease. Metastatic recurrence was noted in ten patients (9.4%). Ten patients (9.4%) were deceased at last follow-up. Of these 10 patients who died, four (3.8%) presented with locally advanced or metastatic disease. Thus, patients who presented with localized paraganglioma of the urinary bladder have a significantly improved survival compared to patients who presented with locally advanced/metastatic disease.

## Discussion

Pheochromocytomas of the urinary bladder are exceedingly rare tumors accounting for less than 0.05% of all bladder tumors and less than 1% of all pheochromocytomas. In the genitourinary tract, the urinary bladder is the most common site for pheochromocytomas (79.2%), followed by the urethra (12.7%), pelvis (4.9%), and ureter (3.2%) [82,83]. Furthermore, approximately 10% of all extra adrenal pheochromocytomas are malignant [83]. Since this is such a rare condition, limited, large reports are available to direct clinical decision making. We extensively reviewed the English literature on this subject and report the largest analysis of pheochromocytomas involving the urinary bladder. Our study improves upon previous large reviews [5,84]. For example, Tsai *et al.* includes one study dating back to 1911, and then eleven studies between 1989 and 2000 [5]. Our study includes 80 studies between 1980 and 2012, offering a better view for analyzing contemporary outcomes. In addition, we have a total of 106 patients included in our study, the most of any literature review to date, while Tsai *et al*. only reported on 53 patients. Lastly, our review used a multitude of demographics to depict the disease process of bladder pheochromocytomas, including presenting symptoms, tumor functionality, tumor size, treatment modality and outcomes.

Symptoms reported in the current literature range from the typical micturition attacks of headache and palpitations to more abstract signs such as paraesthesias and dyspnea. While select patients may lack more common presenting symptoms of bladder pheochromocytoma, *e.g*., hypertension, others may develop hematuria and lower urinary tract symptoms, testifying to the variability in which this disease can present itself. Furthermore, the consequences of hypertension itself may muddle the initial diagnostic picture of these patients. Patients often seek medical attention only when their hypertension has become so advanced as to cause syncope, retinopathy, or intracranial hemorrhage [85]. Physicians must constantly be wary of an undiagnosed pheochromocytoma in the setting of unexplained hypertension or associated symptoms.

Pheochromocytomas can be treated in a number of ways, including catecholamine blockade, surgery, chemotherapy, and radiation therapy. The standard treatment modality for localized or locally advanced pheochromocytomas is surgery, while metastatic or recurrent tumora are treated with palliative therapy [86]. Furthermore, physicians must be aware of poor prognostic indicators, such as large tumor size, advanced stage (≥T3), multifocal tumors, DNA ploidy, and CgA expression [84]. The cyclophosphamide, vincristine, and dacarbazine (the Averbuch protocol) has been shown to be effective against advanced malignant pheochromocytoma [87]. Radiation therapy with ^131^I-MIBG radiation therapy has been used with good efficacy for the treatment of MIBG-avid metastases [88]. Approximately 70% of patients included in our literature review underwent partial cystectomy as a means of primary treatment. Including those who underwent radical cystectomy, over 80% of patients were initially treated with aggressive surgical excision. Of the 75 patients with reported follow up, 15 (20%) experienced recurrence or metastasis at the time of last follow-up, illustrating that good symptomatic control and lower morbidity can be achieved with the surgical resection. It is important to note, however, that 4 of these 75 patients (5.3%) did have recurrence or metastases that ultimately caused their death. In the face of metastatic pheochromocytoma, surgical treatment is rarely curative. It may, however, adequately prolong survival by reducing comorbid conditions (*i.e*. hypertension) and reducing tumor burden, but adjunct therapies are usually indicated [89]. Thus patients should be counseled according to their individual presentation and disease status.

Furthermore, with a lack of high quality data and the lack of organizational guidelines (*e.g.,* EAU, NCCN and AUA) on post-operative follow-up, we recommend no follow-up studies in patients with benign, localized disease. In patients with functional tumors, regardless of stage, VMA, metanephrine and catecholamines levels should be monitored within one month post-surgery, then every six months for two years. Furthermore if regional or metastatic is documented then axial imaging of the abdomen/pelvis should be performed every three months for one year, then every six months for one year, then yearly for three years.

The main limitations of our study relate to its retrospective nature and the large disparity among reporting styles of various institutions. For example, the vast majority of case reports included in this study report on patients presenting with localized or regional disease rather than metastatic disease. This may represent a bias toward reporting on patient’s who’s therapeutic timeline can better be used as an educational platform. The lack of uniformity on how oncologic cases are presented makes it difficult to characterize the true disease course of bladder pheochromocytoma. Information such as patient race, diagnostic findings, and laboratory values should be included whenever possible to better illustrate the pathophysiologic process. Currently, there is no standard staging system for pheochromocytomas. The National Cancer Institute at the National Institute of Health recommends dividing patients into three categories: localized, regional, and metastatic disease. In terms of grade and cellular classification of these tumors, the NCI identifies four pathologic features associated with malignancy: large tumor size, increased number of mitosis, DNA aneuploidy, and extensive tumor necrosis [86]. The complete tumor stage and grade are vital additions to any oncologic case report. Describing these characteristics and developing standard reporting criteria will enable future investigations to better collect and analyze information from case reports.

## Conclusions

In summary, pheochromocytomas of the urinary bladder tend to be functional and occur mostly in young adult Caucasians. Initial presentation is extremely varied in these cancers, necessitating a low threshold of suspicion in the face of hypertension or hematuria. Patients with localized tumors have a favorable prognosis and may be managed by less radical modalities, whereas patients with metastatic disease have a significantly reduced survival rate. Moving forward, it would be helpful to standardize the reporting guidelines of pheochromocytomas cases to better understand the natural process and outcomes.

## Abbreviations

AUA: American Urological Association; VMA: vanillylmandelic acid; H&E: Hematoxylin and eosin; IHC: Immunohistochemical

## Competing interests

The authors declare that they have no competing interests.

## Authors’ contributions

JAB, BS: Acquisition of data, statistical analysis and drafting manuscript. AL, MD: Pathologic interpretation of case report and acquisition pathologic images. JH, MD: Analysis of data and drafting of manuscript. CJR, MD, MBA: Study concept and design, drafting of manuscript. All authors have read and approved the final manuscript.

## Pre-publication history

The pre-publication history for this paper can be accessed here:

http://www.biomedcentral.com/1471-2490/13/22/prepub
